# Early Support after Exposure to Trauma (EASE): protocol for a hybrid effectiveness-implementation trial of an internet-based intervention for PTSD prevention

**DOI:** 10.1186/s13063-026-09502-z

**Published:** 2026-02-05

**Authors:** Espen Rasmussen Lassen, Marianne Skogbrott Birkeland, Karina Egeland, Lise Eilin Stene, Dorte Brodersen, Belinda Ekornås, Nils Petter Reinholdt, Egil Kjerstad, Admassu N. Lamu, Gregory A. Aarons, Erika L. Crable, Maria Bragesjö, Harald Bækkelund

**Affiliations:** 1 Norwegian Centre for Violence and Traumatic Stress Studies, Oslo, Norway; 2 Department of Psychology, University of Oslo, Oslo, Norway; 3 Research Institute, Modum Bad Psychiatric Hospital, Vikersund, Norway; 4 Regional Resource Centre on Violence, Traumatic Stress and Suicide Prevention – Region East (RVTS East), Oslo, Norway; 5 Department of Health and Social Sciences, Norwegian Research Centre (NORCE), Bergen, Norway; 6 Department of Psychiatry, University of California San Diego, 9500 Gilman Drive (0812), La Jolla, CA 92093-0812 USA; 7 Child and Adolescent Services Research Center, 6165 Greenwich Drive, San Diego, CA 92122 USA; 8 Department of Clinical Neuroscience, Centre for Psychiatry Research, Karolinska Institutet, & Stockholm Health Care Services, Stockholm, Sweden; 9 ACTRI Dissemination and Implementation Science Center, University of California San Diego, La Jolla, CA USA; 10 Department of Health Policy and Management, Jonathan and Karin Fielding School of Public Health, University of California, Los Angeles, Los Angeles, CA USA; 11 Center for Health Policy Research, University of California, Los Angeles, Los Angeles, CA USA

**Keywords:** Prevention, PTSD, Psychosocial support, Cost-effectiveness, Implementation, EPIS, CBT-T, CIPE

## Abstract

**Background:**

Post-traumatic stress disorder (PTSD) can have detrimental effects for those afflicted and is associated with increased health care utilization and substantial societal costs. Thus, there is a need for accessible, effective, and cost-efficient preventive interventions for post-traumatic psychological sequelae. Research indicates that trauma-focused cognitive-behavioral therapy (CBT-T) could effectively prevent PTSD when applied as an indicated secondary prevention. CIPE is a scalable, low-threshold, therapist-assisted digital CBT-T, which could be readily implemented in services delivering psychosocial support after traumatic incidents if proven effective and cost-effective. The Early Support after Exposure to Trauma (EASE) study evaluates the effectiveness, cost-effectiveness, and implementation of Condensed Internet-delivered Prolonged Exposure (CIPE), applied as an indicated secondary prevention in the context of Norwegian municipal crisis teams.

**Methods/design:**

The EASE study is a hybrid Type 1 effectiveness and implementation trial. The effectiveness trial is a parallel two-armed multicenter randomized controlled add-on superiority trial, enrolling individuals who receive support from psychosocial crisis teams within 7 weeks after trauma. Participants are randomized to CIPE + treatment as usual (TAU) or TAU only. The primary outcome is the level of PTSD symptoms 6 weeks after randomization (10–13 weeks post trauma). Secondary outcomes include symptoms of depression and insomnia, quality of life, and CIPE cost-effectiveness. The implementation trial examines policy-level factors influencing CIPE implementation, using the Exploration, Preparation, Implementation, Sustainment framework.

**Discussion:**

This study will guide further research, policy shaping, and clinical initiatives for implementing preventive interventions aimed at reducing post-traumatic psychological sequelae by integrating evidence-based interventions into routine psychosocial services.

**Trial registration:**

ClinicalTrials.gov NCT06592677. Registered on 10.09.2024.

**Supplementary Information:**

The online version contains supplementary material available at 10.1186/s13063-026-09502-z.

## Structured summary

**Table:** 

Item	Description
Primary Registry and Trial Identifying Number {4}	The trial is registered with ClinicalTrials.gov Identifier: NCT06592677. Date: 10.09.2024.
Secondary Identifying Numbers	Na
Source(s) of Monetary or Material Support	The EASE study has received funding from the Research Council of Norway (project #344341). The study underwent full external peer review as part of the funding application and process.
Primary Sponsor and contact information {3b}	Norwegian centre for violence and traumatic stress studies. Pb. 181 Nydalen, 0409 Oslo, Norway.
Role of sponsor and funder {3c}	The sponsor covers indemnity insurance and legal liability for the current study, in addition to research infrastructure (e.g., personnel expenses, secure data storage, and data collecting systems). This study is designed and initiated by the PI and CoPIs. Thus, the funding organization (Research Council of Norway) has no role in the formulation of hypotheses, study design, data collection, analyses, or in the drafting of the manuscript.
Contact for Public Queries	Early Support after Exposure to Trauma (EASE) - NKVTS EnglishHarald Bækkelund: Principal investigator. Norwegian centre for violence and traumatic stress studies, Oslo, Norway. harald.bakkelund@nkvts.no
Contact for Scientific Queries	Harald Bækkelund: Principal investigator. Norwegian centre for violence and traumatic stress studies, Oslo, Norway. harald.bakkelund@nkvts.no
Public Title	Early Support after Exposure to Trauma (EASE)
Scientific title	Early Support after Exposure to Trauma (EASE)
Countries of Recruitment	Norway
Health Condition(s) or Problem(s) Studied	F43.0 Acute stress reaction; F43.1 Post-traumatic stress disorder
Intervention(s)	Intervention arm: Condensed Internet delivered Prolonged Exposure (CIPE) + treatment as usual in municipal psychosocial crisis teams Comparator: Treatment as usual only in municipal psychosocial crisis teams
Key Inclusion and Exclusion Criteria	Inclusion Criteria: Receives support from a municipal crisis team • Exposure to a traumatic event (as defined by criteria A for the diagnosis of post-traumatic stress disorder (PTSD) in the DSM-5) within the last seven weeks before randomization • A total score of 10 or above on the PTSD Symptom Checklist-5 at the time of randomization • Age 16 or above • Written informed consent • Writes and speaks English and/or Norwegian Exclusion Criteria: • Severe psychopathology in need of specialized health care (e.g., psychotic symptoms, or high suicide risk) or substance dependence syndrome in need of specialized health care • Known or evident severe cognitive impairment • Ongoing traumatization, violence, or threats • Unstable dose of psychotropic medication two weeks prior to randomization • Concurrent therapy elsewhere before randomization,
Study Type	A hybrid Type 1 effectiveness and implementation randomized controlled trial.RCT design: parallel two-armed multicenter randomized controlled superiority trial, designed to randomize participants to either CIPE + treatment as usual (TAU) or TAU only.
Date of First Enrollment (planned)	September 15th 2024.
Sample Size	Power analysis: N = 150
Primary outcome(s)	PTSD Checklist for DSM-5 (PCL-5)
Key Secondary outcome(s)	- Clinician-Administered PTSD Scale for DSM-5 (CAPS-5) - Patient Health Questionnaire (PHQ-9) - Insomnia Severity Index (ISI) - Client Satisfaction Questionnaire (CSQ-8) - The EuroQol 5‑Dimension 5‑Level questionnaire (EQ-5D-5L) - The Recovering Quality of Life (ReQoL) - Traumatic grief inventory self report (TGI-SR+)
Ethics Review	The trial is approved by the Regional Committee for Medical and Health Research Ethics (REK-sør-øst, #708906) on 23.05.24, and is conducted following guidelines of good clinical practice (GCP) in accordance with the principle of the Declaration of Helsinki. All participants provide written informed consent before enrollment.
Individual Trial Participant Data sharing statement	In accordance with the ethical approval, deidentified individual clinical trial participant-level data is available from the trial PI upon reasonable request.

## Background

The world is currently experiencing a deteriorating security situation characterized by increasing conflicts and wars. Climate change is expected to further escalate the frequency of disasters affecting individuals, families, and societies. The recent COVID-19 pandemic caused significant trauma worldwide, while civilian trauma from domestic abuse and suicide continues to rise in many societies. This convergence of threats underscores the critical importance of societal health preparedness as a priority for nations and multilateral bodies.

Traumatic experiences represent a substantial cause of psychiatric disorders and poor mental health outcomes. A recent nationwide Swedish cohort study provides compelling evidence that traumatic stressors such as injury, assault, and bereavement lead to substantially elevated risks of subsequent psychological disorders, even after controlling for familial factors [1]. Notably, the first year following the traumatic event emerges as an especially vulnerable period, highlighting that psychosocial support during this critical time window must be integrated into all preparedness plans.

Posttraumatic stress disorder (PTSD) is the most prevalent psychiatric condition following traumatic experiences, affecting an estimated 1–9% of individuals over their lifetime [2, 3]. PTSD is associated with significant distress and increased risk of comorbid somatic and psychiatric conditions [4–6], as well as functional impairment, increased healthcare utilization, and substantial societal costs [7].

However, the heterogeneity of victim needs after traumatic experiences presents a significant challenge for societal service planning. While acute stress symptoms—such as increased psychophysiological activation, anxiety, and intrusive memories or flashbacks—are common during the first months following potentially traumatic events (PTE) [8], only approximately one in five individuals exhibit sustained or delayed-onset posttraumatic stress symptom (PTSS) trajectories. The majority demonstrate resilience or natural recovery [9]. Consequently, the United Kingdom’s National Institute for Health and Care Excellence (NICE) guidelines recommend watchful waiting in the aftermath of PTEs to identify those who develop persistent reactions before implementing interventions [10].

Despite this cautious approach, both basic and clinical research indicate that processes occurring within the first weeks to months after a PTE may be decisive for PTSD development, suggesting that preventive interventions could be beneficial during this transitional period [11–14]. Unfortunately, evidence for the efficacy of early preventive interventions after PTEs remains limited. A recent systematic review and meta-analysis reports the strongest support for cognitive-behavioral therapy with a trauma focus (CBT-T) applied as an indicated secondary preventive intervention [15]. The pooled effect sizes of CBT-T range from small to moderate (*SMD* = 0.49–0.70), depending on the control condition employed [15]. Based on this evidence, the International Society for Traumatic Stress Studies’ practice guidelines recommend providing immediate information and support after PTEs, rapidly followed by CBT when indicated [16].

Nevertheless, to our knowledge, CBT-T is not systematically implemented or routinely offered as an early intervention following PTEs in any health system. This gap may reflect the substantial resources required to establish and maintain such treatment capacity, as well as the limited high-quality evidence supporting such investments. Hence, there is an urgent need for low-threshold CBT-T interventions with proven efficacy and cost-effectiveness that can be readily implemented in services delivering psychosocial support after traumatic incidents.

Condensed Internet-delivered Prolonged Exposure (CIPE) represents a promising solution to this challenge. CIPE is an indicated preventive intervention delivered soon after PTE exposure, designed to reduce acute stress symptoms and prevent PTSD development [17–19]. The treatment is delivered as a written, therapist-assisted intensive intervention, with total therapist time typically ranging from 1 to 3 h throughout the program. In a recent randomized controlled trial (*N* = 102), CIPE significantly reduced PTSS compared to a waiting list control [17]. The between-group effect size was moderate at week 3 (*d* = 0.70) and large at week 7 (*d* = 0.83), with effects maintained at the 6-month follow-up and no severe adverse events reported. A qualitative follow-up study with a subsample (*N* = 11) reports that participants describe CIPE as effective, demanding, feasible, and tolerable [18]. These findings suggest that CIPE could represent an effective and cost-efficient approach to indicated PTSD prevention.

However, demonstrating clinical effectiveness and cost-effectiveness is only the first step toward widespread implementation. The translation of evidence-based interventions into routine practice also requires careful consideration of the organizational and policy contexts in which they will be delivered. In Norway, responsibility for psychosocial support after PTEs is assigned to local municipalities, as mandated by various policy documents and laws [20]. However, these policies provide limited specific guidance, resulting in considerable variation in how services are organized and delivered across municipalities. Most municipalities have established psychosocial crisis teams that are activated during crises and disasters to support those affected. These teams typically consist of professionals from diverse backgrounds who work in other services but are called upon during emergencies. Despite growing evidence for effective interventions that can be delivered following a PTE, systematic implementation of such interventions remains lacking in service delivery.

Health policies can function as implementation determinants (i.e., factors that may be barriers or enablers), implementation strategies, or characteristics of the intervention being implemented [21]. In this context, policy serves as an important contextual determinant that influences implementation outcomes [22]. Unclear or conflicting policies may significantly impact crisis teams’ capacity and opportunities to implement evidence-based interventions such as CIPE. As identified in the Exploration, Preparation, Implementation, Sustainment (EPIS) framework, by examining the implementability of CIPE through the lens of EPIS inner (e.g., municipality, crisis services) and outer context (e.g., national, county) factors, innovation characteristics (e.g., CIPE), and bridging factors (i.e., bi-directional influences between outer and inner contexts) [23–25], we can better understand how existing policies influence the implementation of such interventions. This type of analysis can inform the development of targeted recommendations and guidelines to facilitate large-scale implementation, scale-up, and sustainment of evidence-based trauma interventions in Norwegian municipalities.

### Aims and hypotheses

The study hypotheses and research questions are presented in Table 1. The primary aim is to examine the effectiveness of CIPE, delivered approximately 1 to 2 months after a trauma, as an indicated secondary prevention aimed at reducing PTSS and the incidence of PTSD. We will compare the effect of CIPE + treatment as usual (TAU) vs. TAU only, to examine whether CIPE has the potential to improve the ordinary psychosocial services provided by crisis teams. In such contexts, TAU (see “TAU” under the section “Interventions”) is considered to be an appropriate comparator with several benefits [26, 27]. TAU reflects the current real-world practice of the psychosocial crisis teams against which any added benefit of CIPE must be demonstrated, in order to evaluate if CIPE improves standard care and should be routinely implemented in Norwegian municipalities. The study was designed as an add-on trial (i.e., CIPE as an add-on) due to practical and ethical considerations.

**Table 1:** Hypotheses and research questions in the EASE study

Hypotheses concerning the effectiveness and cost-effectiveness of CIPE	
H1	Participants receiving CIPE + TAU will have significantly less PTSS than participants receiving TAU at 6 weeks post T1, and at 6 and 12 months after the traumatic incident
H2	Significantly fewer participants receiving CIPE + TAU will fulfill the criteria for PTSD compared to participants receiving TAU, at 6 and 12 months post trauma
H3	Participants receiving CIPE + TAU will have significantly less symptoms of depression and insomnia than participants receiving TAU at 6 weeks post T1, and at 6 and 12 months after the traumatic incident
H4	Participants in the CIPE + TAU-condition will report significantly higher treatment satisfaction at post-treatment, compared to those in the TAU-condition
H5	Participants with traumatic loss receiving CIPE + TAU will have significantly less symptoms of prolonged grief than participants with traumatic loss receiving TAU 12 months after the loss
H6	Fewer participants in the CIPE + TAU-condition will be referred to second-tier specialty mental health services, and more will achieve improved quality of life within the first year after the traumatic incident, compared to participants in the TAU-condition
H7	The CIPE + TAU implementation is more cost-effective compared to TAU in the short run and may even dominate TAU in the long run (i.e., more effective and less costly)
Research questions concerning the implementability of CIPE	
RQ1	How do existing policies across multi-level outer (i.e., national, county) and inner (i.e., municipality, crisis services) contexts influence implementation of CIPE?
RQ2	What kinds of policy- and systems-level bridging factors are needed to support implementation and sustainment of CIPE?

*CIPE* Condensed Internet-Delivered Prolonged Exposure, *H* Hypothesis, *PTE* potentially traumatic experiences, *PTSD* post-traumatic stress disorder, *PTSS* post-traumatic stress symptoms, *RQ* research question, *TAU* treatment as usual

Secondary aims concerning the effectiveness of CIPE include examination of whether CIPE + TAU is superior to TAU in preventing symptoms of insomnia and depression at 6 weeks post randomization, and 6 and 12 months after a PTE. Further, secondary aims concern the cost-effectiveness of CIPE. Specifically, this study will examine whether CIPE reduces demand for second-tier specialty mental health services and improves quality of life compared to TAU. EASE will also examine if the implementation of CIPE is cost-effective in a long-term perspective, including the effect on labor market participation. The final secondary aims concern the implementation of CIPE. The study will investigate how existing policies across outer and inner context-level influence implementation of CIPE, and what kinds of policy- and systems-level bridging factors are needed to support implementation and sustainment of CIPE. The EASE study design and data collection framework provide the opportunity to examine the extent of long-term mental health needs following traumatic events and to identify predictors of chronic post-traumatic difficulties. Although specific research questions addressing these longitudinal outcomes remain to be developed, such analyses represent potential avenues for future investigation within the project.

## Method

### Research context

Twenty-one municipal crisis teams from the central-eastern parts of Norway participate in the study. The crisis teams are chosen to deliver the intervention due to their aforementioned role in the Norwegian health care system and the well-organized psychosocial services already offered by these teams. Participation requests were directed to municipal crisis teams by the Regional Resource Center for Violence, Traumatic Stress, and Suicide Prevention (RVTS East) to ensure variation in municipal population size, and municipalities from both rural and urban areas. A list of study sites is available in the trial registration (ClinicalTrials.gov identifier: NCT06592677).

### Trial design

**Fig. 1:**
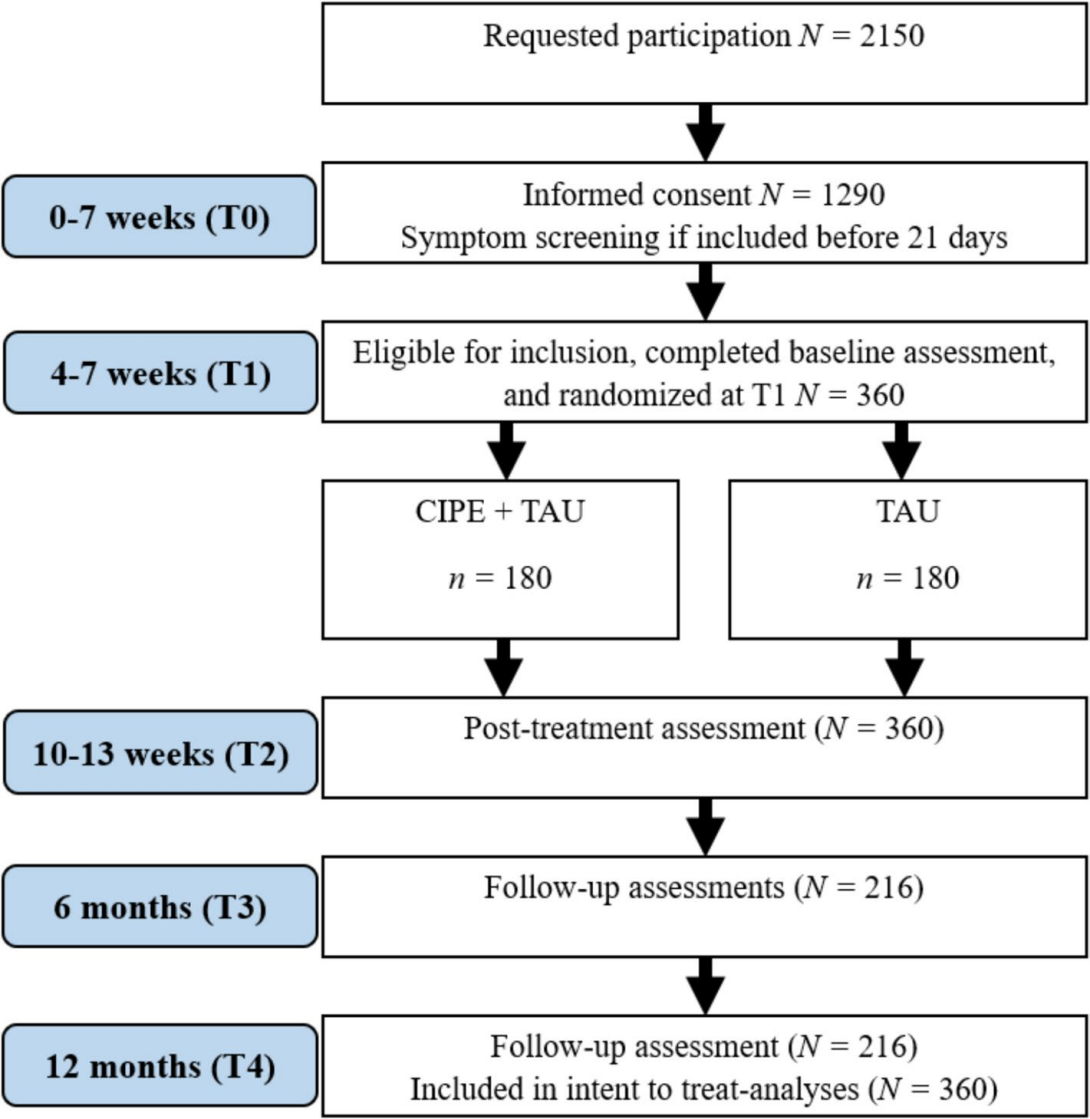
Flowchart of estimated participants in the trial

The EASE study is a hybrid Type 1 effectiveness and implementation trial [28] (for implementation, see section “implementation trial”). The first part of the study is a parallel two-armed multicenter randomized controlled add-on superiority trial, designed to randomize participants to either CIPE + TAU or TAU only (ratio 1:1). All participants will be offered ordinary crisis team services throughout the trial period. The CIPE group will be given CIPE as an add-on between T1 (4–7 weeks after trauma, dependent on the time of enrollment) and T2 (10–13 weeks after trauma, dependent on the time of T1 [i.e., T2 is always 6 weeks after T1]; see Fig. 1). For those included within 3 weeks from the incident, a screening assessment is performed at the time of recruitment (T0). Baseline assessment and evaluation of inclusion and exclusion criteria will be completed at T1. Outcome measures are gathered 6 weeks post randomization (T1), and follow-up measurements are accomplished at 6- and 12-month post PTE. This trial follows the SPIRIT guidelines and methodology [29] (see Additional file 1 for the SPIRIT checklist).

### Inclusion and exclusion criteria

Inclusion criteria are (a) persons that receive support from a municipal crisis team, after (b) exposure to a traumatic event (as defined by criteria A of the PTSD diagnosis in the DSM-5) [30] within 4 to 7 weeks before T1, (c) a total score of 10 or above on the PTSD Symptom Checklist (PCL-5) [31] at T1 (4–7 weeks post trauma, dependent on the time of inclusion), (d) age 16 or above, (e) written informed consent, and (f) writes and speaks Norwegian and/or English.

Exclusion criteria are (a) severe psychopathology (e.g., psychotic symptoms, or high suicide risk) or substance dependence syndrome in need of specialized health care (harmful use is not an exclusion criteria, as long as participants commit to not being under the influence when completing the intervention), (b) known or evident severe cognitive impairment, (c) ongoing traumatization, violence, or threats, (d) unstable dose of psychotropic medication 2 weeks prior to T1, and (e) concurrent therapy elsewhere at the time of T1.

The therapists in the current trial are crisis team health care workers in the included sites (most commonly nurses and social workers). All therapists received training in study procedures. Those delivering CIPE were required to undergo training in CIPE (see the “Therapist training and fidelity” section).

### Recruitment

**Fig. 2:**
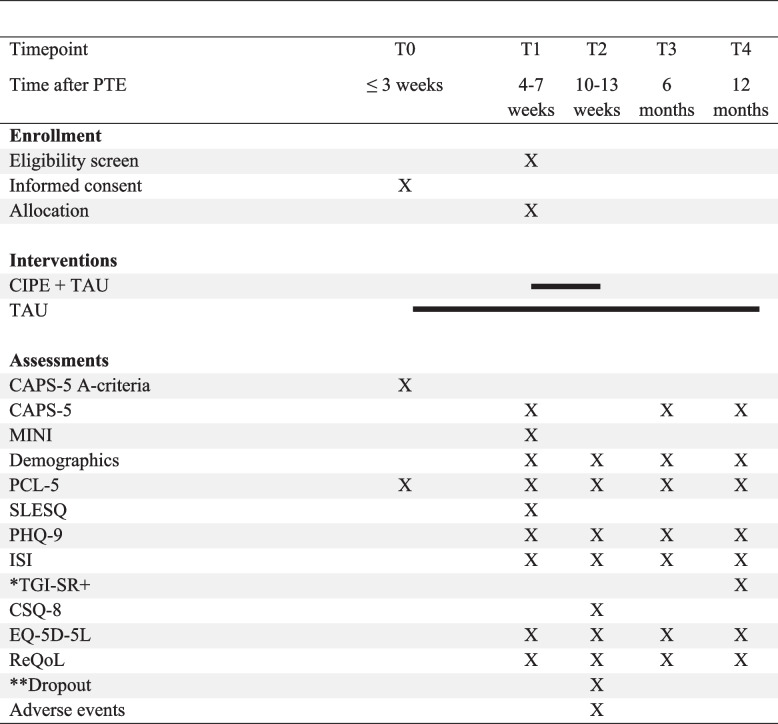
Schedule of enrollment, interventions, and assessments *Note*. CAPS-5: The Clinician-Administered PTSD Scale for DSM-5; CAPS-5 A-criteria: only the assessment of whether the A-criterion is fulfilled in the CAPS-5; CIPE: Condensed internet-delivered Prolonged Exposure; CSQ-8: Client Satisfaction Questionnaire; ISI: Insomnia Severity Index; MINI: The Mini-International Neuropsychiatric Interview version 6; PCL-5: PTSD Symptom Checklist for DSM-5; PHQ-9: Patient Health Questionnaire-9; PTE: Potentially Traumatic Experiences; ReQoL: Recovering Quality of Life; SLESQ: The Stressful Life Events Screening Questionnaire; TAU: Treatment as Usual, TGI-SR+: The Traumatic grief inventory-self report plus.*Administered only to participants who receive support from the crisis team due to traumatic loss.**Administered in the CIPE+TAU-arm only

Figure 2 presents the trials’ enrollment, intervention, and measurement schedule. Participants receive brief information regarding the trial in consultation with the crisis teams, within the first 7 weeks after exposure to a PTE. If consenting, the participants receive exhaustive information about the study, along with a participation request from the research group. Written informed consent is gathered electronically before enrollment and stored in BASS. Crisis team staff repeatedly assure the participants that they will receive TAU, regardless of participation. This is also underscored in all written recruitment material.

At enrollment, the crisis teams and the research group decide whether inclusion criterion B is met, using the Clinician-Administered PTSD Scale for DSM-5 (CAPS-5) [32] A-criterion assessment. If eligible by self-report measures at T1, the participants are assessed with the Mini-International Neuropsychiatric Interview (MINI) [33] version 6 and the CAPS-5 by trained graduate clinical psychology students. The interviews are completed by telephone. ERL (licensed clinical psychologist and PhD candidate) monitors the process. The estimated flow of participants throughout the study phases is visualized in Fig. 1.

### Compensation

To bolster response rates and diminish attrition, participants are given a symbolic monetary incentive [34, 35] in the form of lottery tickets (value: 8€) upon completing all required self-report measures and interviews. This procedure was also approved as a part of the REK approval of the entire trial (see the “Declarations” section).

### Randomization

Eligible participants are randomized 1:1 in random block sizes of 4, 6, or 8 to either CIPE + TAU or TAU only, using the Sealed Envelope software [36] at T1. To mitigate the risk of contamination across treatment conditions, participants from the same household are cluster-randomized. The randomization is stratified on the level of municipal crisis teams to ensure an even distribution of cases in each treatment condition across municipalities. An independent, blinded senior researcher is responsible for all randomization procedures. The allocation is revealed to the study coordinator only after the T1 assessment and decision of inclusion are completed.

### Interventions

#### CIPE

CIPE is an adaptation of Prolonged Exposure (PE) [37]. PE is an exposure-based version of CBT-T that has been thoroughly empirically validated as a treatment for PTSD [38, 39]. CIPE consists of four written therapist-assisted modules, with corresponding homework assignments [17]. The assignments comprise central treatment components, as well as questions regarding essential intervention components and principles, to ensure socialization. Participants are expected to work on these assignments for 6 h weekly and are encouraged to have daily contact with their therapist via a secure email within the treatment platform. Phone sessions are also available when necessary.

#### TAU

Participants in the TAU condition receive standard psychosocial support that is routinely provided by the crisis teams. This could include a varying number of consultations (typically 1–4) consisting of psychoeducation and normalization of symptoms of acute stress, activation of the victim’s social network, practical support, and/or grief support [20]. Although common practice is to follow the guidelines from the Norwegian Directorate of Health [20], there is considerable heterogeneity in what constitutes TAU within and between different crisis teams. This applies both to the content and frequency of support. There are no available detailed descriptions (scientific, official statistics, or other available information) of what TAU constitutes within or across services. Thus, consultation frequency, duration, type, and mode are assessed (see the “Other collected data” section).

### Therapist training and fidelity

The therapists in the current trial are crisis team health care workers. They attend a 1-day seminar held by the originator of CIPE (Bragesjö) or the study PI. The seminar consists of a thorough lecture on the theoretical basis of, and every intervention included in CIPE, as well as practical skills training in CIPE interventions. All therapists receive the CIPE treatment manual (unpublished). As all CIPE treatment components are delivered through standardized text, therapist drift is not possible other than in the secure emails. Continuous monitoring of therapist fidelity in these emails is thus accomplished by two senior consultant clinical psychologists, as well as the trial PhD/clinical psychologist (all trained in CIPE).

### Contamination

For organizational and practical reasons, the majority of the crisis team therapists deliver both TAU and CIPE. Consequently, there is a risk of contamination across conditions at the therapist level. To reduce the risk of contamination, therapists are repeatedly instructed and reminded not to mix the conditions, and are prohibited from using the CIPE internet platform outside of the timeframe of CIPE delivery in each case. Cases of potential contamination will be reported, as the clinicians are instructed to report such cases to ERL.

### Data collection

Figure 2 presents the trials’ enrollment, intervention, and measurement schedule. Participants enrolled within 3 weeks after the incident are assessed with self-report measures at the time of enrollment (T0). All participants are subsequently assessed before randomization (T1), post-treatment (T2), and at 6-month (T3) and 12-month (T4) follow-ups. The main outcome will be measured at post-treatment (T2). Self-report data from participants are collected using an electronic survey solution offered by BASS at the Karolinska Institute, Stockholm. The same system is used to obtain written informed consent and to deliver CIPE. Links to questionnaires are sent to responders using SMS and email. Data from the therapists are collected using Nettskjema and Services of sensitive data (TSD) at the University of Oslo (see “data management and storing”). The research coordinator monitors the data collection process and sends reminders to reduce attrition rates.

### Assessments

#### Primary outcome measure

The PTSD Symptom Checklist (PCL-5) [31] is a 20-item questionnaire that measures DSM-5-specified symptoms of PTSD. The total score ranges from 0 to 80, with higher scores indicating more severe symptoms. Items are rated on a 0 (“not at all”) to 4 (“extremely”) Likert-type scale. The PCL-5 has satisfactory psychometric properties in various trauma populations [40–42]. This study administers a validated Norwegian version of the PCL-5 [43, 44]. As the index trauma is only a few days before T0 for most participants, the recall period is revised to “since the traumatic event” for this measurement. The recall period is “the last week” at T1, and “the last month” at T3 and T4. PCL-5 constitutes the main outcome of this study.

#### Secondary outcome measures

The Clinician-Administered PTSD Scale for DSM-5 (CAPS-5) [32] is a 30-item structured interview, developed to assess current and lifetime PTSD diagnosis, as well as past-week PTSD symptoms. The instrument measures the 20 DSM-5 PTSD symptoms, in addition to the onset and duration of symptoms, related subjective distress, and functional impairment (social, occupational), amongst other aspects of the disorder. Standardized questions and probes are provided. A Norwegian version of the CAPS-5 [45] is administered at T1 and T3–4. CAPS-5 has excellent psychometric properties [32, 46]. The recall period is 1 week at T1, and 1 month at T3 and T4.

The Mini-International Neuropsychiatric Interview (MINI) [33] version 6 is a brief, structured clinician-administered interview designed to assess 17 DSM/ICD diagnoses. Standardized questions and probes are provided. A Norwegian translation of the MINI [47] is administered at T1. The interview has good psychometric properties [33, 48, 49].

A Norwegian version [50] of the Stressful Life Events Screening Questionnaire (SLESQ) [51] is administered at T1 to measure self-reported lifetime exposure to traumatic events. The respondents are asked to report exposure to 15 categories of traumatic events (“yes” or “no”), age at the time of the incident, as well as frequency and duration of the events. Other aspects of the incidents are reported, as well. The Norwegian version of the SLESQ incorporates two extra items compared to the original 13-item version: “Have you ever been directly affected by a natural disaster?” and “Has anyone outside your family, such as fellow pupils or colleagues, repeatedly ridiculed you, put you down, ignored you, or told you were no good?”. The SLESQ has satisfactory validity and reliability [51, 52].

The Patient Health Questionnaire (PHQ-9) [53] is a 9-item self-report measure of depressive symptom severity. The recall period is 2 weeks. Response is given on a 4-point Likert-type scale (0: not at all, 1: several days, 2: more than half the days, and 3: nearly every day), resulting in a total score ranging from 0 to 27. Higher scores indicate higher symptom severity. The instrument is sensitive for both clinical and sub-clinical levels of depressive symptoms and has good psychometric properties [53, 54]. We administer a validated Norwegian version of the PHQ-9 [55].

The Insomnia Severity Index (ISI) [56] is a 7-item questionnaire that measures insomnia symptoms during the past 2 weeks. The instrument utilizes a 5-point Likert-type scale ranging from 0 to 4, yielding a total score of 0–28. Higher scores indicate more insomnia symptoms. The ISI has good psychometric properties [56–58]. A Norwegian version of the ISI is applied.

The Client Satisfaction Questionnaire (CSQ-8) [59] is an eight-item measure of participants’ service satisfaction. Response is given on a scale ranging from one to four, with a resulting total score ranging from 8 to 32. Higher scores suggest higher service satisfaction. A validated Norwegian version of the CSQ-8 is applied [60]. The CSQ-8 is adapted to the context of psychosocial crisis teams in the current trial (e.g., “treatment” adapted to “support”, “therapy” adapted to “support”).

The Traumatic Grief Inventory-Self Report Plus (TGI-SR+) [61] is a 22-item self-report questionnaire measuring symptoms of DSM-5 persistent complex bereavement disorder (PCBD) and ICD-11 Prolonged grief disorder (PGD) in participants who experienced traumatic bereavement. Response is given on a 5-point Likert-type scale ranging from 1 (“never”) to 5 (“always”). The total score ranges from 22 to 110, with higher scores indicating more severe symptoms. The instrument, including the Norwegian version applied here, has good psychometric properties [62, 63]. The instrument is administered at a 12-month follow-up and applies a recall period of the past month.

The EuroQol 5‑Dimension 5‑Level Questionnaire (EQ-5D-5L) [64] assesses self-reported health-related quality of life. It has five items: self-care, mobility, pain/discomfort, usual activities, and anxiety/depression, each measured on a 5-level severity scale (no, slight, moderate, severe, or unable/extreme problems). The instrument defines 3125 possible health states, valued by a representative sample of the general Norwegian population using the hybrid valuation method—composite time-trade-off and discrete choice experiment [65]. The utility scores are anchored on a − 0.453—1 scale, with 1 indicating “full-health,” 0 equivalent to “being dead,” and values below 0 are worse than being dead. The EQ-5D-5L data are used to generate quality-adjusted life year (QALY) used for cost-utility analysis (CUA). The instrument has excellent psychometric properties [66].

The recovering quality of life (ReQoL) is an 11-item outcome measure assessing psychopathology-specific quality of life in mental health contexts [67]. For each item, response is given on a 5-point scale (never to most of the time or always). The study also uses the ReQoL utility index (ReQoL-UI), which is a recovery-focused generic preference-based measure derived from ReQoL. ReQoL-UI constitutes six mental health items (activity; belonging; choice, control, and autonomy; hope; self-perception; and well-being) and a physical health item, each with five severity levels. The ReQoL-UI defines 78,125 different health states valued by a representative member of the UK public using a conventional time-trade-off method [68]. The ReQoL-UI is also used to generate QALYs for CUA in mental health interventions. The ReQoL-UI values range between − 0.195 (for the worst health state) and 1 (for the full-health state), with zero indicating a health state equivalent to being dead.

#### Other collected data

A structured adverse events (AE) questionnaire applied in the aforementioned RCT of CIPE [17] is utilized. The questionnaire was used in a previous trial with similar results to face-to-face interviews [69]. The instrument captures the frequency, duration, and nature of possible adverse events, and such events are further classified based on their perceived association with CIPE (related or unrelated). Both participants and clinicians are asked to report and rate participants’ short- and long-term discomfort of any AEs on a scale from 0 (“did not affect me at all”) to 3 (“affected me very negatively”).

In both TAU and CIPE, all time and resource use of therapists (i.e., treatment, travel, online tasks, appointments, preparation, and competence level of therapists) are registered. Registry data is also utilized for all participants, using the Norwegian Patient Register (NPR), Control and Payment of Health Reimbursement (KUHR), Municipal patient- and user-register (KPR), and FD-Trygd. However, in the absence of reliable registry data on the use of health care resources in the municipalities, this study applies survey data reported every second month by the crisis teams’ personnel, via a generic questionnaire. Consultation duration, type (e.g., CIPE, grief support, practical support), and mode (e.g., telephone, video consultation, home visit), as well as the professional background of the involved personnel, are reported from T1 to T4.

In the CIPE + TAU-arm, both clinicians and participants are assessed with a nine-item generic, unvalidated self-report measure of reasons for drop-out from CIPE, in cases where the participant did not finish CIPE. The questionnaire maps lack of motivation, disliking of e-therapy, lack of relevance, lack of reduction of PTSS, worsening of PTSS, remission, concurrent therapy elsewhere, and other (qualitative response category) as possible sources of drop-out. Response is given in one of two categories (0: no, 1: yes), except for the mentioned qualitative item.

Participants are also asked to self-report sociodemographic and background data: year of birth, gender, relationship and cohabitation status, country of birth (self and parents), educational and employment status, current use of psychotropic medication, current psychological treatment (outside of the trial), and ongoing threats or violence.

### Statistical analyses

H1–3 are tested with linear mixed effects models. The linear mixed effects model includes fixed effects for treatment group (intervention vs. control) and time (post-intervention, 6 and 12 months). In a hierarchical setup, a second step includes a treatment × time interaction. Random effects include random intercepts for participants to account for individual differences. The mixed effects models are applied using the R package nlme [70]. H4–6 are examined using independent t-tests. The quality-of-life change (change in QALY) of participants in CIPE vs. TAU (H6) is analyzed using the mixed effects model. Estimation is conducted using restricted maximum likelihood, with all available observations included, yielding unbiased estimates under the assumptions of missing at random or missing completely at random. For the t-tests, missing data are handled using multiple imputation.

Hypothesis 7 involves a comprehensive economic evaluation comparing the cost-effectiveness of CIPE relative to TAU in terms of both costs and consequences/effectiveness [71]. Costs are assessed using the ingredients approach, which involves three steps: first, identifying each resource required for the intervention; then, measuring these resources; and finally, valuing them. The effectiveness of the intervention is measured by QALY—a composite measure combining both quantity of life (mortality) and quality of life (morbidity) into a single metric [72]. For each arm, mean costs and mean QALYs are estimated across all participants to calculate the incremental cost (ΔC) and incremental effect (ΔQALY). The incremental cost-effectiveness ratio (ICER) or incremental cost per incremental QALY (ΔC/ΔQALY) is then calculated for decision-making. To accept the intervention, ICER should be less than the willingness-to-pay or cost-effectiveness threshold. Since there is no official threshold in Norway, we apply a threshold recommended by the Norwegian Directorate of Health (NOK 500 000 at 2005 prices), adjusted to current price levels [73].

This economic evaluation is conducted in two ways: trial- and model-based approaches. In the former, we compare trial-specific intervention costs and effects. Following recommendations by NICE [74], we perform a cost-utility analysis combining intervention-related and wider healthcare costs with changes in QALYs. In the latter, we develop a decision analytic model to simulate the long-term (lifetime) cost-effectiveness of CIPE. The model extrapolates beyond the trial period to estimate the potential lifetime costs and QALY outcomes under different assumptions. In both approaches, model parameter uncertainty is assessed using both probabilistic and deterministic sensitivity analyses.

### Power and sample size calculations

Previous CIPE studies report effect sizes within the range of *d* = 0.6–0.8. For the primary hypothesis in this study, a more conservative effect size of *d* = 0.3 is expected. With 95% power and an *α*-level of 0.05, 150 participants (73 in each group minimum) are required to detect a significant between-group effect. Consequently, assuming a 20% data attrition rate, at least 183 participants should be recruited. The municipal crisis teams involved in the study currently cover approximately 1,080,000 inhabitants. Although official statistics on the number of traumatic events handled by these teams are lacking, based on the best unofficial statistics available, a conservative estimate suggests 0.5 to 1 case per 1000 inhabitants annually. With a cautious estimate of 0.5, and with each incident involving a mean of two victims aged 16 or older, about 2150 victims are eligible for recruitment over 2 years. Moreover, we assume a ~50% response rate [75] and that approximately 30% have PTSD symptoms 1 month after the incident [76]. These parameters result in a sample of 360 participants, which constitutes the recruitment target. Assuming an overall attrition rate of 40%, 216 participants will complete the 12-month follow-up assessment.

### Oversight and monitoring

#### Data management and storage

Each enrolled participant is assigned a unique study ID. The ID number is linked to each participant’s social security number, stored in a separate coupling key-file in the Services of sensitive data (TSD) at the University of Oslo. TSD uses the highest possible level of IT security, and data files are continuously backed up. Participant self-report data is gathered in BASS (identical security procedures as TSD) and securely exported to TSD for statistical analyses. Trial data are stored at least 5 years after data collection is concluded (anticipated completion date: December 31 st, 2027). A Data Protection Impact Assessment (DPIA) with risk and security measures is in place, collaborating with Sikt (the national agency providing shared digital services and infrastructure for the Norwegian knowledge sector). The Data Protection Official at Sikt and at the Norwegian Centre for Violence and Traumatic Stress Studies (sponsor’s) both acknowledge the DPIA. Only the principal investigators and ERL will have access to the final trial dataset. ANL and EK will have access to the data necessary for the investigation of H6–7. 

#### Safety and monitoring procedures for adverse events

Despite the lack of severe adverse events (AE) in a recent RCT of CIPE [17], the trial has established procedures to monitor AEs and serious adverse events (sAE). Health personnel in the crisis teams are instructed to monitor and address AE continuously. Unmet healthcare needs that are discovered by the research teams in the T1–4 assessments are communicated to the individual’s therapist in the municipality. Moreover, self- and clinician-reported (s)AE are assessed at T2 (see instruments). Adverse events are defined as any unfavorable experience occurring to participants during the trial. Serious adverse events are defined as incidents including death, (attempted) suicide, serious self-harm, acute mania or psychosis, severe intoxication, or other psychological or somatic conditions requiring emergency medical care.

After the first 18 participants are randomized (corresponding to 10% of the target sample size based on the power analysis), an internal pilot study will be conducted in accordance with the CONSORT guidelines for randomized feasibility and pilot trials [77, 78]. The pilot will assess AEs and sAEs. If the rate of AEs is substantially higher than reported in previous studies of CIPE and/or any sAEs occur, the advisory board will consider protocol modifications and/or discontinuation of the trial. Should such deliberations arise, the project owner will retain the final authority to decide whether the trial will be terminated.

### Blinding

The graduate clinical psychology students performing the clinical interviews are blinded. Participants are instructed not to report their assigned condition in interviews. In case of spontaneous unblinding, the interview is terminated and repeated in its entirety by another blinded assessor. Blinding of participants and therapists is not possible due to the nature of the interventions. Data analysis is conducted by the trial statistician (MSB), who is blinded to treatment assignment for the entirety of the trial. ANL and EK perform health economic analyses and are blinded to treatment condition.

### Protocol violations and revisions

All included participants are included in the intent-to-treat analyses. Participants who do not complete CIPE within T2, or report adjustment of psychotropic medication or concurrent therapy elsewhere, as well as ongoing traumatization, violence, or threats after T1, and drop out of the trial, are not included in the completers’ analyses. Protocol revisions have been and will be updated in the clinical trial registry and communicated to REK for approval in accordance with the contract.

### Implementation trial

The overarching aim of the implementation trial is to understand how policy- and systems-level factors across national, regional, and municipal levels influence the uptake and sustainment of CIPE within municipal crisis services operating under multi-level governance. The study maps how national policies, regional agreements, and municipal plans interact to shape CIPE implementation, including considerations of costs and political support, as well as bridging factors such as policy directives, agreements, and/or contracts for services. It assesses how policy guidelines, regional arrangements, and municipal plans align with or diverge from CIPE requirements, with the goal of producing practical recommendations to strengthen bridging mechanisms, promote durable adoption, and inform scaling of CIPE across municipalities.

To examine how existing policies across outer and inner contexts influence CIPE implementation [21, 23, 25], the study adopts a mixed qualitative design that combines document review, qualitative interviews, and observational methods. Key topics across document reviews and interviews include how policies influence service provision, how stakeholders at both policy and system levels (e.g., municipal service providers, policy developers) consider costs, the level of political support for crisis services and CIPE, whether there are ambiguities in responsibility and decision-making, and misalignment between agencies, and organizational culture affecting CIPE uptake.

The document review includes sentinel policy surveillance methods for tracking laws and policies over time regarding the organization of municipal crisis teams. The legal mapping is carried out at three governance levels: national (Ministry and Directorate of Health), county/regional (County Governors or equivalent regional authorities), and municipal (leaders or team members of psychosocial crisis teams). At the national and county levels, relevant policy documents on psychosocial preparedness, follow-up, funding, responsibilities, and intergovernmental collaboration are identified and refined in consultation with the Ministry of Health and Care Services, the Directorate of Health, and the County Administrator. A structured document analysis maps how policies frame preparedness and follow-up, and define roles and accountability across levels. At the municipal level, a stratified sampling of municipalities is carried out to capture diversity in geographic and systemic contexts. Psychosocial preparedness plans are reviewed, focusing on content, organization, providers, context, and collaboration with GPs and referral pathways to specialized services.

The qualitative interviews involve semi-structured interviews with actors at the same three governance levels: national, county, and municipality. At the national and county level, relevant persons who have participated in the formulation of, or are responsible for implementing relevant laws and policy documents at the Ministry of Health and Care Services, the Directorate of Health, and the County Administrator are interviewed. At the municipal level, municipal crisis team leaders or other psychosocial care providers are interviewed to understand which policies guide planning and organization. The output will be a policy map detailing alignment and gaps between national directives and municipal practices. In addition to document reviews and interviews, training materials, formal agreements, and observational notes collected during the CIPE testing are included.

Data are analyzed iteratively and thematically to identify potential bridging factors, barriers, enablers, and how policy contexts and decision-making processes shape implementation choices. A logic model will illustrate how outer context and inner context factors can support sustainable CIPE implementation, including uptake, costs, political support, relational ties (e.g., partnerships), and processes (e.g., communication, data-sharing) and long-term viability. The analysis explicitly addresses bridging mechanisms and will culminate in actionable policy implementation strategies and recommendations for durable intergovernmental collaboration (contracts, partnerships, data-sharing).

### Dissemination

The study results will be published in scientific journals irrespective of whether the results support the hypotheses or not.

## Discussion

This study has three working packages with respective aims. First, the study aims to investigate the effectiveness of CIPE, as an add-on treatment to TAU in psychosocial crisis teams, in reducing post-traumatic psychological sequelae. Second, the cost-effectiveness of CIPE is examined. Third, the influence of existing policies on the implementation and sustainment of CIPE is researched.

Although most individuals exposed to a PTE do not develop PTSD, a substantial minority experience prolonged post-traumatic sequelae. There is promising meta-analytical evidence for indicated secondary preventive interventions based on CBT-T interventions [15], and such early interventions are efficacious and safe [17, 79]. This trial adds to the literature by examining the effectiveness of CIPE on reducing post-traumatic psychological distress in the context of Norwegian municipal crisis teams. Consequently, the trial could generate important knowledge regarding which individuals are at risk of developing PTSD after PTEs, and whether CIPE effectively reduces post-traumatic psychological distress.

Beyond clinical outcomes, the trial generates knowledge about the economic implications of implementing CIPE, including potential reductions in the need for specialist secondary mental health care. Furthermore, EASE investigates the implementation of preventive interventions for PTSD in primary-level health services such as municipality crisis services. Additionally, we examine how health policies impact the implementation of evidence-based practices in this context. Both these research topics are understudied in implementation science [21]. In summary, the EASE study addresses important knowledge gaps concerning evidence-based secondary prevention of PTSD and psychosocial care for victims of crises and disasters, and the health economics and implementation factors involved in such interventions.

### Strengths and limitations

The current study has several strengths. First, the multi-center effectiveness design improves the external validity, providing policy makers and municipal crisis teams with important knowledge regarding evidence-based indicated preventive interventions after trauma. If CIPE is effective in reducing PTSS within the context of municipal crisis teams, this could enhance the generalizability of the findings significantly, as the implementation closely reflects how the crisis teams typically operate. Second, EASE represents an innovation in research on early interventions after PTEs and hybrid trial designs, by investigating the effectiveness, cost-benefits, and implementability of CIPE in a single trial. Finally, the use of a scalable, digitalized, evidence-based indicated preventive intervention (i.e., CIPE) in ordinary services (i.e., not ad-hoc services established after major disasters) is another strength.

This study also has limitations that warrant consideration. First, the therapists and participants are not blinded, which could induce placebo effects. Nevertheless, the interviewers are blinded, ensuring single-blind assessments. Furthermore, there might be unequal treatment intensity across the groups (i.e., the TAU + CIPE condition receiving more treatment than the TAU-only condition). There is also a risk of contamination between conditions due to the aforementioned design. Moreover, recruitment could be delayed due to multiple factors (e.g., low response rates, participant preference for avoidance instead of exposure, study implementation barriers on therapist or system level).

### Trial status

Protocol version 3.0 (18.09.2025) is currently active. The recruitment is ongoing since the 15th of September 2024. The estimated recruitment completion date is September 2026. The estimated project completion date is December 31, 2027.

## Supplementary Information

Additional file 1.

## Data Availability

The datasets generated during and/or analysed during the current study are not publicly available, following the ethical approval, but deidentified individual clinical trial participant-level data is available from the trial PI on reasonable request. Only the principal investigators and ERL will have access to the final trial dataset. ANL and EK will have access to the data necessary for the investigation of H6-7.

## Abbreviations

AE: Adverse events
CAPS-5: The Clinician-Administered PTSD Scale for DSM-5
CAPS-5 A-criteria: Only the assessment of whether the A-criteria has been fulfilled in the CAPS-5
CBT: Cognitive-behavioral therapy
CBT-T: Cognitive-behavioral therapy with a trauma focus
CIPE: Condensed Internet-Delivered Prolonged Exposure
CSQ-8: Client Satisfaction Questionnaire
CUA: Cost-utility analysis
DPIA: Data protection impact assessment
DSM-5: Diagnostic and Statistical Manual for Mental Disorders, fifth edition
EPIS: Exploration, Preparation, Implementation, Sustainment Framework
EQ-5D-5L: EuroQol 5-Dimension 5-Level Questionnaire
H: Hypothesis
ICER: Incremental cost-effectiveness ratio
ISI: Insomnia Severity Index
KPR: Municipal Patient- and User-Register
KUHR: Control and Payment of Health Reimbursement
MINI: Mini-International Neuropsychiatric Interview version 6
NICE: National Institute for Health and Care Excellence
NPR: Norwegian Patient Register
PCL-5: PTSD Symptom Checklist for DSM-5
PE: Prolonged exposure
PHQ-9: Patient Health Questionnaire-9
PTE: Potentially traumatic events
PTSD: Post-traumatic stress disorder
PTSS: Post-traumatic stress symptoms
QALY: Quality-adjusted life year
ReQoL: Recovering quality of life
ReQoL-UI: ReQoL Utility Index
RQ: Research question
(s)AE: Severe adverse events
SLESQ: Stressful Life Events Screening Questionnaire
TAU: Treatment as usual
TGI-SR+: Traumatic Grief Inventory-Self Report Plus
